# Smoking, alcohol consumption, and illicit substances use among adolescents in Poland

**DOI:** 10.1186/s13011-018-0179-9

**Published:** 2018-11-29

**Authors:** Maria Nowak, Malgorzata Papiernik, Alicja Mikulska, Bozena Czarkowska-Paczek

**Affiliations:** Department of Clinical Nursing, Medical University of Warsaw, E. Ciolka Street 27, 01-445 Warsaw, Poland

**Keywords:** Alcohol use, Cigarette use, Illicit substance use, Parental acceptance of abuse, Adolescents

## Abstract

**Background:**

The use of alcohol, tobacco, and illicit substances typically first occurs in adolescence. The purpose of this study was to examine alcohol, cigarette, and illicit substance use among adolescents in Poland, including the age of initiation, frequency of use, methods of access, location of use, and parental knowledge and attitude.

**Methods:**

An author-derived questionnaire was used to cross-sectionally survey 541 participants aged 13–17 years old. Due to the fact that some answers were lacking, the number of questionnaires analysed was 538 in case of smoking and illicit substances use, and 535 in case of alcohol drinking.

**Results:**

The use of alcohol, cigarette, and illicit substance among the investigated group was 36.1, 37.6, and 10.8% respectively. The average age of initiation was 13–14 years old. Parents were aware of alcohol, cigarette, and illicit substance use 49.5, 35.8 and 22.4% of the time, respectively, and the rate of acceptance was 5.7 and 6.7% for alcohol and cigarettes. More than 28% of participants smoked in school, and 32.7% accessed illicit substances in the school’s neighborhood.

**Conclusions:**

The rate of alcohol, cigarette, and illicit substance use in Poland is high and increasing, despite globally designed preventative actions. Parents’ awareness of children’s alcohol, cigarette, or illicit substance use is low, and schools hardly fulfil their educational and protective role. Preventative actions are necessary, and local challenges should be considered.

## Background

The use of alcohol, tobacco, and illicit substances typically first occurs in adolescence. Adolescents usually start with cigarettes and alcohol, and these substances often precede the subsequent use of illicit substances. In addition, the close relationship between cigarettes smoking and the use of alcohol and drugs has been established in the literature [1–3]. It has been reported that people who start smoking and alcohol drinking in adolescence have an increased probability of having substance use problems in adulthood [4, 5]. Drinking itself, especially initiated in childhood, is associated with a wide range of negative outcomes including suicide ideation, violence, injury, depression, and school absenteeism [6]. Alcohol use in men is strongly associated with the use of physical force to have sex [7]. Exposure to high levels of nicotine during adolescence may result in damage to sensitive developmental phases and may have a cumulative effect over time. Strong et al. [8] showed, that early cigarette smoking among African Americans is correlated with later educational problems and long-term unemployment. Daily smoking increases the likelihood of remaining or becoming physically inactive over time [9]. Adolescents who report consistent drinking and smoking have higher rates of deviant behavior and violence, and are more likely to have problems with the law in their 20s [10].

The social and health care costs resulting from the cigarette smoking are very high and increasing [11]. Despite the fact that smoking and alcohol consumption are preventable causes of death and disease, they still contribute to decreased life expectancy among men and women [12]. Because smoking and illicit substances-related problems are the function of the duration and intensity of use, preventive activity, directed especially towards adolescents, is of critical concern [13, 14]. The prevalence and usage of alcohol, cigarettes, and illicit substances vary by sex and geography [4, 6]. Therefore, the current situation in individual regions regarding alcohol, cigarettes, and illicit substance use should be examined to establish the optimal strategy for prevention.

The aim of this study was to examine the use of alcohol, cigarettes, and illicit substances among adolescents in Poland and to extrapolate conditions and habits associated with this usage in younger age groups.

## Methods

This study used a cross-sectional survey design in which adolescents completed the questionnaire on one occasion. All questions used in the questionnaire were author-derived.

The study was approved by the Bioethics Committee of the Medical University of Warsaw (No AKBE/187/2017).

In total, 541 adolescents aged 13–17 years old (mean 14 ± 1 years) enrolled in the study. The investigation was performed in gymnasia in towns of the county of Mazovia, Poland with varying number of inhabitants: below 20,000 (42.5% (*n* = 230) of enrolled participants), between 100,000–200,000 (34.2% (*n* = 185) of enrolled participants) and above 200,000 (23.3% (*n* = 126) of enrolled participants).

Each participant received a cover letter explaining the purpose of the study and ensuring confidentiality. The names of responders were not recorded on the questionnaire, thus rendering the data anonymous. Informed consent was indicated by voluntary participation in the survey. Completion of the questionnaire was done with previous instruction performed by the investigators. The collection rate was 100%.

The questionnaire was divided into three parts. Each part included questions investigating alcohol, cigarette, and illicit substance use: the age of initiation, current use, frequency, the kind of illicit substances, access to these substances, places of consumption, and parent’s knowledge and attitude towards the potential abuse.

Due to the fact that some answers were lacking, the number of questionnaires analysed was 538 in case of smoking and illicit substances use, and 535 in case of alcohol drinking.

## Statistical analysis

The statistical analysis was performed using SPSS. Descriptive statistics of the collective data were generated using standard statistical parameters: percentage, mean, and standard deviation. Differences between females and males were analysed using either the Mann-Whitney U test or Pearson’s chi-squared test. Differences between residents of towns with different numbers of inhabitants were analysed using either the Jonckheere-Terpstra test or Pearson’s chi-squared test. The Kendall’ tau-b correlation was used to analyse correlation of current use of alcohol, illicit substances and cigarette smoking. Results were considered statistically significant when *p* < 0.05.

## Results

### Alcohol drinking

The total number of analysed questionnaires was 535. According to this survey study, lifetime prevalence of alcohol included 54.4% (*n* = 291) of participants, and currently 36.1% (*n* = 193) drink alcohol, most of them less than once a month (*n* = 133, 68.9%), but some (*n* = 31, 16.1%) drink daily. The average age of first alcohol consumption was 13–14 years old (*n* = 98, 18.3%), but for 10.3% of participants (*n* = 55) it was younger than eight years old. Among those who drink alcohol, 42.3% (*n* = 82) indicated that it was supplied by someone older, 38.7% (*n* = 75) bought alcohol themselves, and the rest from other sources. Parents of 49.5% (*n* = 96) of the adolescents were aware of their alcohol consumption, 25.5% (*n* = 49) accepted alcohol drinking at family celebrations, and 5.7% (*n* = 11) accepted it completely.

The Jonckheere-Terpstra test and Pearson’s chi-squared test revealed that in smaller towns more adolescents currently drink alcohol, the average age of their first alcohol consumption was lower, and more parents accepted alcohol drinking than in larger towns (*χ*^*2*^(2, *n* = 535) = 47.60, *T*_*JT*_ = 53,580,50; *z* = 3,61, *χ*^*2*^(4, *n* = 193) = 47.28 respectively, *p* < 0.001 in all cases).

The results referring to alcohol use (the age of initiation, current use of alcohol, frequency of use, and attitude of parents) stratified by sex are shown in Table 1. The average age of first alcohol consumption was lower in males, more males currently drink alcohol, and frequency of drinking in males is higher when compared to females (*p* < 0.001, *p* < 0.001, *p* = 0.001 respectively).

**Table 1:** Alcohol drinking, the age of initiation, frequency of drinking and the parent’s attitude to alcohol drinking among adolescents in Poland stratified by sex

		Sex							*p*
		female		male		total			
		*n*	%	*n*	%	*N*	%		
The age of initiation	Below the age of 8 years	15	5,19	40	16,26	55	10,28	*Z = 4,71*	*< 0,001*
	8–10 years of age	18	6,23	17	6,91	35	6,54		
	11–12 years of age	36	12,46	40	16,26	76	14,21		
	13–14 years of age	52	17,99	46	18,70	98	18,32		
	15–16 years of age	12	4,15	13	5,28	25	4,67		
	17 years of age	0	0,00	2	0,81	2	0,37		
	Never drank alcohol	156	53,98	88	35,77	244	45,61		
	Total	289	100,00	246	100,00	535	100,00		
Current alcohol drinking	I don’t drink alcohol	206	71,28	136	55,28	342	63,93	*χ*^*2*^*(1,* *N* *= 535) = 14,74*	*< 0,001*
	I drink alcohol	83	28,72	110	44,72	193	36,07		
	Total	289	100,00	246	100,00	535	100,00		
Frequency of alcohol drinking [referred only to those, who declared alcohol drinking]	daily	8	9,64	23	20,91	31	16,06	*Z* *= 3,35*	*0,001*
	4 times a week	1	1,20	8	7,27	9	4,66		
	2 times a week	3	3,61	6	5,45	9	4,66		
	Once a week	1	1,20	5	4,55	6	3,11		
	Once a month	2	2,41	3	2,73	5	2,59		
	Occasionally	68	81,93	65	59,09	133	68,91		
	Total	83	100,00	110	100,00	193	100,00		
parental attitude to alcohol drinking [referred only to those, who declared alcohol drinking]	Accept on family celebrations	23	27,71	26	23,85	49	25,52	*χ*^*2*^(2, *N* = 192) = 0,54	0,797
	Accept	4	4,82	7	6,42	11	5,73		
	Don’t accept	56	67,47	76	69,72	132	68,75		
	Total	83	100,00	109	100,00	192	100,00		

Differences between female and male were analysed using either U Mann-Whitney test or Pearson chi-square test. (Those, who currently do not drink alcohol could have the history of alcohol drinking in the past)

### Cigarette smoking

The total number of analysed questionnaires was 538. From the whole investigated group, 39.8% (*n* = 214) of participants had lifetime prevalence of cigarette smoking, and currently 37.6% (*n* = 201) smoke cigarettes, most of them daily (*n* = 111, 55%). The average age of smoking initiation was 13–14 years old (*n* = 106, 19.7%), but for 4.8% of participants (*n* = 26) it was younger than eight years old. From those who smoke cigarettes, 23.9% (*n* = 48) declared that they were supplied with cigarettes from someone older, 30.9% (*n* = 62) bought cigarettes themselves, and 29.9% (*n* = 60) of participant get cigarettes at home because other residents smoke. The rest of the participants indicated other sources of supply. Parents of 35.8% (*n* = 72) of the adolescents were aware of their cigarette smoking, 3.6% (*n* = 7) accepted cigarettes smoking occasionally, 6.7% (*n* = 13) accepted it completely, and 10.3% (*n* = 20) smoked with their children. It was very interesting that 28.9% (*n* = 58) of participants smoked in school, almost the same number as on celebrations (*n* = 56, 27.9%), and more than at home (*n* = 31, 15.4%). The rest of investigated participants declared that they smoke in other places.

Pearson’s chi-squared test revealed that in smaller towns more parents accept adolescent cigarette smoking than in larger towns (*χ*^*2*^(6, *n* = 195) = 13.19, *p* < 0.025).

The results referring to cigarette smoking (age of initiation, current use of cigarettes, frequency of use, and attitude of parents towards smoking) stratified by sex are shown in Table 2. The average age of initiation of smoking was lower in males, and more males than females currently smoke cigarettes (*p* < 0.001 in both cases).

**Table 2:** Cigarette smoking, the age of initiation, frequency of cigarettes smoking and the attitude of parents to cigarette smoking among adolescents in Poland stratified by sex

		Sex							*p*
		female		male		total			
		*n*	%	*n*	%	*N*	%		
The age of initiation	Below the age of 8 years	3	1,03	23	9,27	26	4,83	*Z* *= 4,09*	*< 0,001*
	8–10 years of age	10	3,45	12	4,84	22	4,09		
	13–14 years of age	54	18,62	52	20,97	106	19,70		
	15–16 years of age	8	2,76	9	3,63	17	3,16		
	17–18 years of age	0	0,00	1	0,40	1	0,19		
	Never smoked cigarettes	195	67,24	129	52,02	324	60,22		
	Total	290	100,00	248	100,00	538	100,00		
Current cigarettes smoking	I don’t smoke cigarettes	204	70,59	130	52,85	334	62,43	*χ*^*2*^*(1,* *N* *= 535) = 17,84*	*< 0,001*
	I smoke cigarettes	85	29,41	116	47,15	201	37,57		
	Total	289	100,00	246	100,00	535	100,00		
Frequency of cigarettes smoking [referred only to those, who declared cigarettes smoking]	15–20 cigarettes per day	6	7,06	17	14,66	23	11,44	*Z* = 0,73	0,467
	10–15 cigarettes per day	5	5,88	3	2,59	8	3,98		
	5–10 cigarettes per day	23	27,06	29	25,00	52	25,87		
	1–5 cigarettes per day	11	12,94	17	14,66	28	13,93		
	Occasionally	40	47,06	50	43,10	90	44,78		
	Total	85	100,00	116	100,00	201	100,00		
parental attitude to cigarettes smoking [referred only to those, who declared cigarettes smoking]	Accept occasionally	2	2,47	5	4,39	7	3,59	*χ*^*2*^(3, *N* = 195) = 1,01	0,845
	accept	5	6,17	8	7,02	13	6,67		
	I smoke together with parents	7	8,64	13	11,40	20	10,26		
	Don’t accept	67	82,72	88	77,19	155	79,49		
	Total	81	100,00	114	100,00	195	100,00		

Differences between female and men were analysed using either U Mann-Whitney test or Pearson chi-square test. (Those, who currently do not smoke cigarettes could have the history of smoking in the past)

### Illicit substance use

The total number of analysed questionnaires was 538. From the whole investigated group, 15.8% (*n* = 85) of participants had a lifetime prevalence of illicit substance use, but currently only 10.8% (*n* = 58) actively use those substances, 13.8% (*n* = 8) of them more than three times a week. The average age of initiation of illicit substance use was 13–14 years old (*n* = 56, 10.4%), but for 1.3% of participants (*n* = 7) it was younger than eight years old. From those who use illicit substances, 32.8% (*n* = 19) declared that they were supplied in the school neighborhood, 27.6% (*n* = 16) bought those substances from colleges, and 19% (*n* = 11) were engaged in drug distribution themselves. The rest of the participants indicated other sources of supply. Parents of 22.4% (*n* = 13) of adolescents engaging in illicit substance use were aware of it. It was very interesting that 50% (*n* = 29) of participants use illicit substances at school, almost the same number as in entertainment clubs (*n* = 26, 44.8%). The rest of investigated participants declared that they use these substances in other places.

Most of the investigated participants declared the use of marijuana (*n* = 34, 58.6%), then LSD (*n* = 7, 12.1%), cocaine (*n* = 5, 8.6%), amphetamines (*n* = 3, 5.2%) and other (*n* = 9, 15.5%).

Pearson’s chi-squared test revealed that more adolescents use illicit substances in smaller towns than in larger towns (*χ*^*2*^(2, *n* = 550) = 6.91, *p* < 0.05).

The results referring to the use of illicit substances (age of initiation, current use, frequency of use) stratified by sex are shown in Table 3. The average age of first use of an illicit substance was lower in males, and more males than females currently use those substances (*p* = 0.001 and *p* < 0.001 respectively).

**Table 3:** Illicit substances use, the age of initiation, and frequency of illicit substances use among adolescents in Poland stratified by sex

		Sex							*p*
		female		male		total			
		*n*	%	*n*	%	*N*	%		
The age of initiation	Below the age of 8 years	1	0,34	6	2,42	7	1,30	*Z* *= 3,24*	*0,001*
	11–12 years of age	10	3,45	8	3,23	18	3,35		
	13–14 years of age	19	6,55	37	14,92	56	10,41		
	15–16 years of age	2	0,69	2	0,81	4	0,74		
	Never used illicit substances	258	88,97	195	78,63	453	84,20		
	Total	290	100,00	248	100,00	538	100,00		
Current use of illicit substances	I don’t use illicit substances	272	94,12	207	83,47	479	89,20	*χ*^*2*^*(1,* *N* *= 537) = 15,71*	*< 0,001*
	I use illicit substances	17	5,88	41	16,53	58	10,80		
	Total	289	100,00	248	100,00	537	100,00		
Frequency of illicit substances use [refers to those who declared illicit substances use]	1–2 in life time	2	11,76	7	17,07	9	15,52	*Z* = 0,44	0,658
	Occasionally	11	64,71	25	60,98	36	62,07		
	1–3 a week	1	5,88	4	9,76	5	8,62		
	More than 3 times a week	3	17,65	5	12,20	8	13,79		
	Total	17	100,00	41	100,00	58	100,00		

Differences between female and men were analysed using either U Mann-Whitney test or Pearson chi-square test. (Those, who currently do not use illicit substances could have the history of the use in the past)

Correlations between those who currently drink alcohol, smoke cigarettes, and use illicit substances are shown in Table 4.

**Table 4:** The Kendall’ tau-b correlation between current use of alcohol, cigarettes and illicit substances

	Current alcohol use		Current cigarettes use		Current illicit substances use	
	*τ*_*b*_	*p*	*τ*_*b*_	*p*	*τ*_*b*_	*p*
Current use of alcohol			0,36	< 0,001	0,30	< 0,001
Current cigarettes use	0,36	< 0,001			0,35	< 0,001
Current illicit substances use	0,30	< 0,001	0,35	< 0,001		

## Discussion

Our results undoubtedly reflect that the use of alcohol and cigarettes is high among Polish adolescents, whereas the use of illicit substances is rather low. We did not investigate the reason but hypothesize that it could be cost. It has been proven that the price increase of cigarettes significantly reduced the rate of smoking in different age groups, including adolescents [15]. Another reason could be the support for banning illicit substances among adolescents in Europe, especially high for heroin, cocaine, and ecstasy [16]. Additonally detering might be that the use of illicit substances carries more negative consequences than the use of alcohol or cigarettes.

According to Scheithauer, et al. [17], the highest rates of alcohol use among European adolescents is in Eastern Europe. They also reported huge differences between countries, from 85.7% in Estonia to 21.6% in Iceland. The lifetime prevalence of alcohol consumption among adolescents in Norway reported by Mangerud et al. [4] was 54.4%, and it was higher in females than in males, which is inconsistent with our results. The lifetime prevalence of alcohol use in Poland, as reported by Scheithauer, et al. [17], was 65%. In our study, about 55% of participants reported lifetime alcohol use. This difference might result from the characteristics of investigated group. About 12% of the group investigated by Scheithauer, et al. [17] were required to repeat a grade, which could influence the results. Another reason could be the different size of towns, where the investigation was conducted. It was also shown, that adolescents’ drinking was associated with adolescents’ own socio-economic position [18]. The lifetime prevalence of alcohol use in Poland, as reported in The 2015 ESPAD (European School Survey Project on Alcohol and Other Drugs) Report was 83%, what is more compared to our results. The difference could result from the characteristic of the sample. ESPAD survey was conducted among adolescents who turn 16 in the calendar year of the survey, and all samples were nationally representatives [19].

In 2010, the World Health Organization (WHO) reported that the prevalence of adolescent cigarette smoking in Poland was about 16% in males, and 12% in females. The prevalence of cigarette smoking among Polish adults the same year was 38 and 24% respectively for males and females [20]. According to our data, smoking among adolescents increased compared to the last report from the WHO, and is now higher than the prevalence of smoking reported for adults in 2010. The lifetime prevalence of cigarettes smoking among adolescents in Poland, as reported in year 2015 by ESPAD was 55%, what is more in compare to our results. The difference could result from the aforementioned difference in the characteristics of the sample [19].

Among the WHO regions, Europe has the highest prevalence of cigarette smoking among adults (28%), and some of the highest among adolescents, but it differs across countries. According to the Health Behavior in School-aged Children (HBSC) study, cigarette use ranges from 5 to 51% in young males and from 1 to 53% in young females in Armenia and Greenland respectively. The lifetime prevalence of cigarette smoking among adolescents in Norway reported by Mangerud et al. [4] was 13.2%, and it was higher in females than in males, which is inconsistent with our results. It was also reported that in some countries in Central-Eastern Europe cigarette smoking among adolescents is very similar to that among adults, which is in line with our results [20]. According to the 2010 WHO report, in the years 2000–2010, four tobacco advertising, promotion, and sponsorship restrictions were implemented. It seems that the results of those promotions were poor, and efforts towards reducing adolescent smoking must be retargeted.

According to Scheithauer, et al. [17], the lifetime prevalence of cannabis use in Poland is 15%, and the average age of first use is 13–14 years old. According to ESPAD [19] thelifetime prevalence of cannabis use among adolescents in Poland was 21%. These results are slightly higher than those from our study, however these differences likely results from the aforementioned differences in investigated groups. Illicit substance use also differs across countries, for example, in Norway, the lifetime prevalence of illicit substance use among adolescents was 3.8%, much less than in Poland [4].

The results discussed above differ from those on adolescents in the United States. It is estimated that about 25% of American adolescents drink alcohol, 11% smoke cigarettes, and as much as 20% use illicit substances [21].

It should be mention here, that according to ESPAD [19], the lifetime prevalence of the tranquilizers and sedatives use without prescription among adolescents in 2015 was most prevalent in Poland - 17%, while the average use in all 35 ESPAD countries was 6%. Also the use of anabolic steroids was high in Poland – 3%, while the average use in ESPAD countries was 1%.

Data discussed above clearly show that the use of addictive substances varies by region, country, and defined population. Our study additionally revealed that the use of these substances was higher in smaller towns than larger ones, and that there were sex-based differences; however, these data were partially contrary to data obtained in other countries [4, 17].

There are some aspects of the alcohol, cigarette, and illicit substance use that should be highlighted. First is the age of initiation. In our study the average age of first substance use was 13–14 years old, which for alcohol and illicit substances is in line with results published by Scheithauer, et al. [17]. They also reported that 15 and 2% of responders indicated that their first use of alcohol or cannabis respectively was at younger than ten years old, which is comparable with our results. Due to the fact that negative outcomes related to the use of these substances depend on the duration and intensity of use, the age of initiation should be paramount when considering preventive actions. Realistically, preventative measures should be directed not only to teenagers, but also to elementary school aged children.

Another important issue is the role of parents. The results of our study showed that half of the participants’ parents knew about their alcohol use, and more than 30% accepted it completely or at least at family celebrations. In case of cigarettes the situation was similar, however, fewer parents were aware of it though as much as 10% smoked cigarettes together with their children. The number of parents aware of illicit substance use was even lower than for alcohol and cigarettes. These results reveal that parental awareness of alcohol or cigarette use among children in Poland is lower than other countries in Europe [22]. The parental role is very important, but also very difficult and ambiguous. It was shown that the provision of alcohol by parents is associated with lower levels of harm at the same drinking and bingeing frequency [6, 22]. Children who consumed alcohol without parental knowledge were less likely to report having a parental discussion about alcohol in the last three months or report lifetime receipt of at least one parental protective measure [22]. It is important to acknowledge that the acceptance rate of alcohol and cigarette use is high in Poland, which likely results from many factors, including the lack of sufficient knowledge about associated problems and outcomes. Our results clearly show that preventive actions should also be directed to the parents, and should include information on the harmful outcomes of substance use and the symptoms of this use in children.

Another issue is the role of school in protecting adolescents from substance use in Poland. Our study revealed that between 29 and 50% of participants smoke or use illicit substances at school. A lot of users buy illicit substances in the school’s neighborhood. In general, schools represent an opportune setting for interventions to prevent adolescent substance use because they have access to large number of adolescents and have curricula and other opportunities that promote health and well-being. Our results suggest that the educational and protective role of schools should be strengthened.

Adolescents in Poland have easy access alcohol and cigarettes, despite laws prohibiting the sale of alcohol and cigarettes to people under 18 years old. Trafficking and use of illicit substances is also prohibited by law, regardless of age. The common method of accessing alcohol and cigarettes was through older individuals, which has been previously reported [23]. Surprisingly, notable numbers of underage people report purchasing their own alcohol and cigarettes, at about 39 and 62% respectively. For alcohol, this is higher than in Great Britain, where only 28% of underage drinkers have purchased alcohol themselves [23]. These results showed failure to comply with minimum age legislation in Poland.

Our study confirmed that cigarette and alcohol use share common etiological factors and often develop concurrently [24]. Animal studies have revealed reinforcing interactions between these two substances, which are age and sex dependent with adolescent males being the most sensitive to it. In humans, it is well established in the literature that cigarette use likely increases the vulnerability of teenage boys to alcohol use [5, 25].

Some limitations of the study need to be taken into account. The investigated group did not include adolescents who were not a part of the regular school system. Very important limitation of the present study is relying on self-reported data. Therefore this report might be influenced by information bias, which would have led to an under- or overestimation of cigarette smoking, alcohol drinking and illicit substances use. This was also cross-sectional study, and the temporal nature of associations cannot be excluded.

## Conclusion

According to this survey study, the rate of alcohol, cigarette, and illicit substance use in Poland is high and differs between small and large towns. Parental awareness of alcohol or cigarette use among children in Poland is low, and schools hardly fulfil their educational and protective role. These circumstances should always be considered when planning preventative action. The conclusion from an article by Dwyer-Lindgren, et al. [26] is that public health is local, and information on local challenges is required to increase resident’s and policy maker’s awareness. The article refers to life expectancy and the mortality rate, and their conclusions have universal significance. Alcohol, cigarette, and illicit substance use are responsible for 9% of the global disease burden and 12% of deaths worldwide [4, 27]. Lowering the rates of alcohol, cigarettes and illicit substances use should lower these numbers. Preventative actions must consider to a greater extent the local conditions and challenges, and they should gravitate from universal methods towards more personalized approaches. Our findings provide local information for revising global prevention programs.

## Abbreviations

ESPAD: European School Survey Project on Alcohol and Other Drugs
HBSC: Health Behavior in School-aged Children
WHO: World Health Organization
